# Current Status of Research on the Modification of Thermal Properties of Epoxy Resin-Based Syntactic Foam Insulation Materials

**DOI:** 10.3390/polym13183185

**Published:** 2021-09-19

**Authors:** Zhongyuan Zhang, Xiaohan Dai, Le Li, Songsong Zhou, Wei Xue, Yunpeng Liu, Hechen Liu

**Affiliations:** 1 Hebei Key Laboratory of Distributed Energy Storage and Micro-Grid, North China Electric Power University, Baoding 071003, China; hvzzy_01@163.com (Z.Z.); dxhjy139@163.com (X.D.); liuyunpeng@ncepu.edu.cn (Y.L.); hc.liu@ncepu.edu.cn (H.L.); 2 China Electric Power Research Institute, Beijing 100192, China; z18516979216@163.com; 3 State Network Zhejiang Electric Power Co., Ltd. Integrated Services Branch, Hangzhou 310000, China; xuewei202021@163.com

**Keywords:** syntactic foam insulation materials, composite material, epoxy resin, thermally conductive particles

## Abstract

As a lightweight and highly insulating composite material, epoxy resin syntactic foam is increasingly widely used for insulation filling in electrical equipment. To avoid core burning and cracking, which are prone to occur during the casting process, the epoxy resin-based syntactic foam insulation materials with high thermal conductivity and low coefficient of thermal expansion are required for composite insulation equipment. The review is divided into three sections concentrating on the two main aspects of modifying the thermal properties of syntactic foam. The mechanism and models, from the aspects of thermal conductivity and coefficient of thermal expansion, are presented in the first part. The second part aims to better understand the methods for modifying the thermal properties of syntactic foam by adding functional fillers, including the addition of thermally conductive particles, hollow glass microspheres, negative thermal expansion filler and fibers, etc. The third part concludes by describing the existing challenges in this research field and expanding the applicable areas of epoxy resin-based syntactic foam insulation materials, especially cross-arm composite insulation.

## 1. Introduction

Recently, with the development of materials and technology for electrical equipment, composite insulation materials (insulators, insulation cross-arms) have been used in domestic transformer substations and lines. They have replaced the traditional porcelain insulation materials in AC and DC power systems due to their unique antifouling performance and superior properties. However, these electrical insulation materials have more stringent requirements on inner filling materials because they operate outdoors for many years and bear strong electric fields and erosion by harsh weather conditions, and they are affected by factors such as high and low-temperature changes, wind, rain, and snow. Among them, the composite foam material with porous composite structure is cured by the resin matrix filled with low-density filler. It has attracted significant research attention as filling materials in various composite insulation materials owing to their low density, high strength, and low defects, and their water absorption rate and mechanical properties are far better than those of traditional natural foams.

As a thermosetting resin with high insulation properties, epoxy resin is widely used as the resin matrix for syntactic foams. The molecule of the epoxy resin contains two or more epoxy groups that can form a three-dimensional (3D) cross-linked network under the action of a suitable curing agent. The resulting network exhibits low water absorption, impact resistance and crack resistance. Epoxy-based syntactic foams have been extensively studied in China, some of whose functions can be optimized, or the development of certain unfavorable characteristics can be suppressed, by adding specific functional fillers into epoxy resins [1]. The application of epoxy resin-based syntactic foam in missile launchers can effectively reduce the density and improve the heat resistance of the launcher. Epoxy resin-based syntactic foams can be employed in solid buoyancy materials [2] to effectively reduce their water absorption [3,4], and can greatly improve the compressive strength of lightweight buoyancy materials. This feature is suitable for water diffusion leakage current testing of insulation materials used in electrical equipment. In general, compared with other foams, epoxy resin-based syntactic foams have the following special advantages: (1) They have a very high closed cell rate, and thus excellent electrical insulation. (2) Their base material is epoxy resin, which does not require low-temperature storage conditions as does phenolic foam [5,6]. (3) They have excellent chemical resistance. (4) They are flame retardant as the oxygen index can reach 40% without adding any flame retardant. (5) The preparation process is simple and inexpensive.

**Figure 1:**
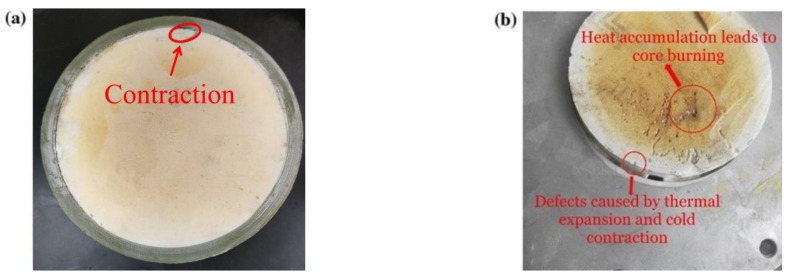
Thermal expansion and core burning of filling material in composite cross-arms (**a**) Cracking of a cross-arm sample; (**b**) Core burning of a cross-arm sample.

There are more stringent evaluation criteria in the application of epoxy resin-based syntactic foams in internal insulation, including higher insulation strength, lower water absorption, higher thermal conductivity, and lower coefficient of thermal expansion (CTE), etc. Take the application of insulation filling in composite insulation cross-arms as an example. On the one hand, poorly matched CTE will cause a large amount of residual stress in the composite cross-arm, resulting in falloff or cracking during the molding process, and these affect the performance and service life of the cross-arm [7,8,9] (Figure 1). On the other hand, due to the low thermal conductivity, it is difficult to transfer the heat generated during the resin cross-linking reaction, which causes the core-burning phenomenon in the composite [10] (Figure 1b). Therefore, there is a need to modify the thermal properties of epoxy-based syntactic foam to improve the thermal expansion and conductivity of syntactic foam insulation materials. Most studies focus on modifying the thermal properties of resin–matrix composites because the research and application of epoxy-based syntactic foam insulation materials is still evolving, and research on the modification of thermal properties is still insufficient. However, epoxy-based syntactic foams are two-phase foam materials and their structure and material properties are different from those of traditional solid composites. Therefore, the results based on traditional solid composites cannot be fully applied to epoxy-based syntactic foam.

This paper reviews the recent progress of the thermal conductivity mechanism, thermal conductivity model and filling modification of epoxy resin-based composite foam thermal conductivity materials, including the filling modification of thermal conductivity particles, hollow glass microspheres (HGMs) and negative thermal expansion (NTE). Finally, the prospect of application of composite foam material promotes the application and development of epoxy resin-based composite foam, which is of great significance to improve the reliability of composite insulation material.

## 2. Thermal Characteristics Mechanism and Model of Composites

### 2.1. Thermal Conductivity of Composite

**Figure 2:**
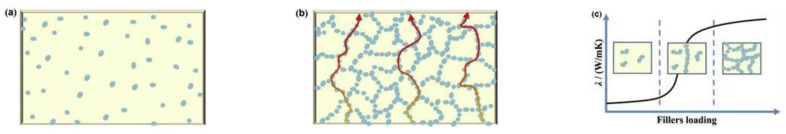
Schematic diagram of heat conduction mechanism (**a**) “Sea Island” under low filler loading; (**b**) Heat conductive path under high filler loading; (**c**) Change in the thermal conductivity of the composite materials. (**a**–**c**) Adapted with permission [16], Copyright 2020, Elsevier Ltd.

The thermal conduction mechanism of polymer composites is mainly divided into two aspects: thermal conduction percolation theory and thermal conductive pathway theory. Herein, we focus on the latter. The thermal conductivity of composites is improved to various degrees by adding thermally conductive fillers into the matrix [11,12]. It is reflected in the “sea-island” structure when the content of the filler is small, which indicates very little contact between the fillers, and the heat transfer is very fast even in the filler. Moreover, even if the heat transfer is very fast in the fillers, the thermal resistance of the filler–matrix interface limits the improvement of the thermal conductivity of composites [13,14]. The addition of fillers reduces the distance between the “sea islands” until the occurrence of contact phenomena. Thus, pathways are formed through some parts or the entire material. Then, the thermal conductivity of the filled composite is significantly improved (Figure 2a). In this process, the agglomeration phenomenon between fillers (which cannot be ignored) can be considered a turning point for constructing thermal conductive network pathways. Once the agglomeration is transformed into a thermally conductive network structure (Figure 2b), the thermal conductivity will increase rapidly (Figure 2c) [15].

### 2.2. Thermal Conductivity Model of Composites

#### 2.2.1. Theoretical Model of Spherical Filler

Maxwell [17,18] obtained the thermal conductivity equation for composites based on spherical fillers by solving the Laplace equation:

(1) $$\lambda =\frac{2\lambda_{1}+\lambda_{2}+2V\left(\lambda_{2}-\lambda_{1}\right)}{2\lambda_{1}+\lambda_{2}-2V\left(\lambda_{2}-\lambda_{1}\right)}\lambda_{1},$$

where λ_1_, λ_2_, and λ are the thermal conductivities of the matrix, filler, and composite, respectively, and V is the volume of the fillers.

This model is suitable for predicting thermal conductivity when a single spherical filler is filled with a volume of less than 1%.

Bruggeman [19] reported that the Maxwell equation can be obtained by adding only a small amount of dV to the composite with higher spherical content. Then, the equation of the composite of the spherical-filler filling system with high filling content can be obtained through integration as follows:

(2) $$1-V=\frac{\lambda_{1}-\lambda }{\lambda_{2}-\lambda_{1}}{\left(\frac{\lambda_{1}}{\lambda }\right)}^{\frac{1}{3}},$$

These two models are suitable for predicting only the binary system of spherical fillers, but when the thermal conductive chain (network) is formed between the filler particles with ultra-high filling content, the Agari equation, given below, must be used [20,21]:

(3) $${\mathrm{lg}\lambda =V}_{f}C_{2}{\mathrm{lg}\lambda }_{2\;}+\left(1-V_{f}\right)\mathrm{lg}\left(C_{1}\lambda_{1}\right),$$

where C_1_ is the factor that affects the crystallinity and size of the polymer crystal, λ_1_, λ_2_, and λ are the thermal conductivities of the matrix, filler, and composite, respectively, and V_f_ is the volume of the fillers.

#### 2.2.2. Theoretical Model of Fiber Filler

The Springer–Tasi semiempirical model is proposed based on the assumption that the filler is cylindrical fiber and distributed at right angles in the matrix. The thermal conductivity formula is given as:

(4) $${\lambda =\lambda }_{1}\{1-2\sqrt{\frac{V}{\pi }}+\frac{1}{B}[\pi -\frac{4}{\sqrt{1-\left(\frac{B^{2}V}{\pi }\right)}}\tan^{-1}(\frac{\sqrt{1-(\frac{B^{2}V}{\pi })}}{1+\frac{B^{2}V}{\pi }})]\},$$

where B = 2(λ_1_/λ_2_) − 1 is the semiempirical model.

#### 2.2.3. Theoretical Model of Flake Filler

The Hatta model is a formula specially used to predict the thermal conductivity of composites filled with flake fillers [22], and it is given as follows:

(5) $$\frac{\lambda }{\lambda_{1}}=1+\frac{V}{S\left(1-V\right)+\frac{\lambda_{1}}{\lambda_{2}-\lambda_{1}}},$$

where S is a physical quantity related to the direction of the thermal conductivity measurement. When the thermal conductivity of a material is measured along a plane, S = πL/4X, and when measured along the thickness, S = 1 − (πL/2X), where L is the effective diameter of the flake filler in the composite, and X is the thickness of the flake filler in the composite.

#### 2.2.4. Theoretical Model of Irregular Filler

Wang [23] improved the Bruggeman equation based on irregular particle shape, particle content, and interface thermal resistance as follows:

(6) $${(1-V_{2})}^{n}=(\frac{\lambda_{1}}{\lambda_{2}}{)}^{(1+\mathrm{na}-a)(1-a)}{[\frac{\lambda_{c}-\lambda_{2}(1-a)}{\lambda_{1}-\lambda_{2}(1-a)}]}^{n/(1-a)}$$

#### 2.2.5. Theoretical Model of Multifiller

When one type of thermally conductive filler cannot meet the requirement for actual applications, the above-mentioned thermal conductivity model cannot be used. A new model that can be applied to the number of filler particles in the system is established based on previous research work as follows:

(7) $${\mathrm{lg}\lambda =V}_{f}\left(X_{2}C_{2}{\mathrm{lg}\lambda }_{2}{+X}_{3}C_{3}{\mathrm{lg}\lambda }_{3}\ldots \ldots \right)+\left(1-V_{f}\right){\mathrm{lgC}}_{1}\lambda_{1}$$

where λ_1_, λ_2_, and λ are the thermal conductivities of the matrix, filler, and composite, respectively, V_f_ is the volume fraction of the mixed filler in the composite material, and X_i_ is the percentage of each filler in all fillers.

### 2.3. Thermal Expansion Mechanism of Composite

**Figure 3:**
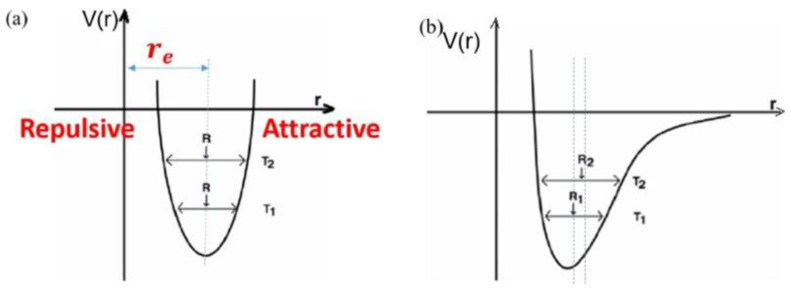
Relationship between the potential energy of (**a**) harmonic and (**b**) nonharmonic motion and atomic distance.

CTE is an important indicator for quantifying the expansion or contraction deformation of a material after being heated. The thermal expansion of a solid can be summarized as follows: when the temperature increases, the kinetic energy of adjacent atoms in the solid increases, increasing the average distance and the generation of thermal expansion. This is the source of vibration for thermal expansion. When the degree of separation of atoms is symmetrical to the potential energy, zero thermal expansion occurs, and NTE is the same as shown in Figure 3 [23]. In Figure 3a, the ordinate V(r) is the potential energy of the interaction between atoms, and the abscissa r is the size of the interaction distance between the atoms. The interaction force between atoms is expressed as (−(dE(r))/dr). The equation shows that when the potential energy corresponding to the r_e_ position is the smallest, and the force is 0, which indicates the most stable structure. When the temperature increases, the interaction force between two atoms can be completely offset, and the equilibrium position does not change with the temperature; therefore, there is no macroscopic thermal expansion. In normal circumstances, atoms cannot return to equilibrium (that is disharmonic vibration), which is macroscopically expressed as positive thermal expansion or NTE, as shown in Figure 3b. When materials with different CTEs are used to control the CTE of composites, it can be realized in terms of material ratio and microstructural design. When combined with actual fillers, it can be further subdivided into the concentration, size, and shape of the filler.

### 2.4. Thermal Expansion Model of Composites

The thermal expansion of materials can be characterized by CTE, that is, the relative change in volume per unit temperature can reflect the degree of thermal expansion of a material [24].

CTE of most solid materials is in the order of 10^−6^. The thermal expansion of anisotropic materials differs in different directions; therefore, the linear CTE needs to be used to express them. Under constant pressure, the coefficient of linear expansion α_l_ is defined as:

(8) $$\alpha_{l}=(\frac{\partial \mathrm{lnl}}{\partial T}{)}_{P}=\frac{1}{l}{\left(\frac{\partial l}{\partial T}\right)}_{P}\;\approx \;\frac{1}{l}\frac{l-l_{0}}{T-T_{0}}=\frac{1}{l}\frac{\Delta l}{\Delta T}\;$$

where l is the length along the main axis.

For a material with volume V, when the change in volume after a temperature change of ΔT is ΔV, then CTE can be defined as:

(9) $$\alpha_{V}=(\frac{\partial \mathrm{lnV}}{\partial T}{)}_{P}=\frac{1}{V}{\left(\frac{\partial V}{\partial T}\right)}_{P}\;\approx \;\frac{1}{V_{0}}\frac{V-V_{0}}{T-T_{0}}=\frac{1}{V_{0}}\frac{\Delta V}{\Delta T}\;$$

where V and V_0_ are the volume under pressure.

For isotropic materials, such as cubic structures, the expansion coefficients along each axis of the crystal are equal; i.e., α_V_ = 3α_l_. For anisotropic materials, such as hexagonal and tetragonal structures, the expansion coefficients of the crystals along each axis are different; α_l_ is not related to α_V_.

In addition, the Gruneisen equation can also be used to express the relationship between the CTE of a material and temperature [25].

This is expressed by the mode Gruneisen equation as:

(10) $$\alpha_{V}=\frac{1}{\mathrm{BV}}\overset{3N}{\underset{i=1}{\sum }}C_{i}\gamma_{i}\;$$

and by the average Gruneisen parameter as:

(11) $$\alpha_{V}=\frac{C_{V}\overset{-}{\gamma }}{\mathrm{BV}}$$

where α_V_ is the coefficient of volume expansion, T the temperature, γ the Gruneisen constant, C_V_ the specific heat capacity of the object, and B the elastic modules of the object.

## 3. Modification of Thermal Characteristics of Epoxy Resin Composite Foam Insulation Materials

There are two main techniques to improve the thermal performance of insulating composites. The first is to change the body structure of the matrix, thereby improving the thermal conductivity and thermal expansion performance of the matrix [26,27,28]. However, this method cannot meet the actual production and application requirements because the polymer synthesis is complicated, and the cost of the matrix material is high. The second method is improving the thermal performance of epoxy-based composite foam by adding functional fillers. Compared with the first method, this method is easy to perform and suitable for mass production, and it is also a research hotspot worldwide. This article reviews the epoxy-based foam materials considering filler modification, including the addition of thermally conductive particles, HGM, negative CTE materials, and fibers.

### 3.1. Filling Modification of Thermally Conductive Particles

**Figure 4:**
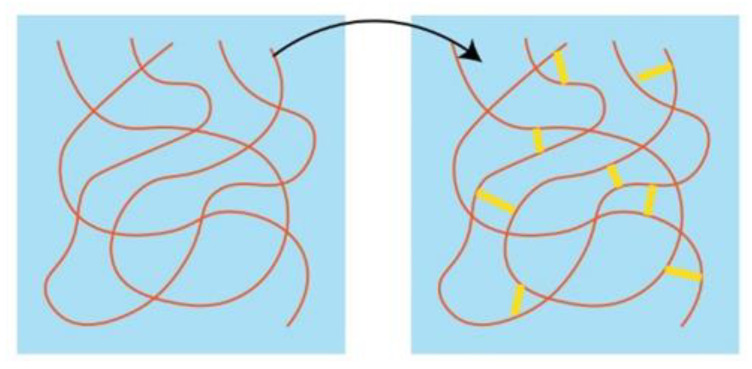
Schematic of the interaction between engineering molecules of heat transfer between chains. Adapted with permission [29], Copyright 2021, Nature Materials.

There are two factors affecting the thermal conductivity of composites filled with thermally conductive particles. The first is the heat conduction pathway, that is, the ability to form phonon transmission paths. If a heat conduction filler can form more heat conduction channels in a composite, the phonon transmission capacity would be stronger, and the thermal conductivity of the composite would be higher. However, most of the thermally conductive particles form chains or agglomerates inside the matrix. When the thermal conduction chain structure is generated, the weak interaction between the chains becomes thermal resistant. Therefore, the capacity for heat transmission of the polymer can be improved by enhancing the interconnection between the chains, which increases the thermal conductivity of the polymer. Then, the internal structure of the polymer is as shown in Figure 4 [29].

**Figure 5:**
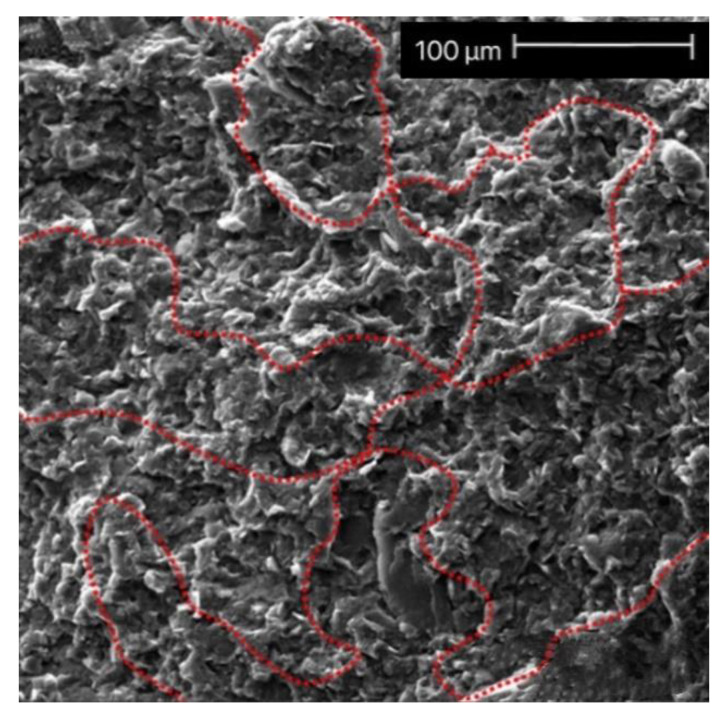
SEM image of the thermal path formed by the connection of thermally conductive fillers (Mag = 500×). Adapted with permission [30], Copyright 2018, Advanced Composites and Hybrid Materials.

When the agglomeration occurs, the thermal conductivity of the composite is not greatly improved, but it is considered a turning point in the construction of thermal conduction network pathways. Once the agglomeration is transformed into a thermally conductive network structure, the thermal conductivity increases rapidly. The SEM image of a thermally conductive path formed by a connected thermally conductive filler is shown in Figure 5. The second factor affecting the thermal conductivity of composites filled with thermally conductive particles is the interface thermal resistance. The thermal resistance between the matrix molecules is generally much greater than that between fillers. If there are more filler–matrix interfaces, it is difficult to increase the thermal conductivity of the composite. After combining the above factors with the actual filler, the factors affecting the thermal conductivity of composites can be further subdivided into the concentration, size, shape, type of filler, and interaction between the matrix and the filler [1,29,30,31,32]. Chung et al. [33] studied the effects of filler content, particle size, oxygen content, and surface treatment on the thermal conductivity of epoxy-based composites. Within the range of factors investigated in the research, it was found that the filler content has the greatest impact on the thermal conductivity of composites. As the proportion of filler volume was increased from 10% to 60%, the thermal conductivity increased by 1567%. Among the other factors, particle size had the greatest impact on the thermal conductivity of composites. When the filler content was 60%, the increase in the percentage of thermal conductivity reached at 106%, which is attributed to the particle size.

Epoxy resin can be varied in different degrees by adding different fillers to the epoxy group, which not only reduces the cost but also expands the application range of the composite. Adding some common fillers with high thermal conductivity and low thermal expansion can significantly improve the thermal performance of epoxy-based composites. Huang et al. [34] used silver nanoparticles to add polyvinylidene fluoride to test its effect on thermal conductivity. When the loading was higher than 15.0%, the thermal conductivity of the composite increased significantly. When the loading reached 20.0%, the thermal conductivity was about 27 times that of pure polyvinylidene fluoride. Datsyuk et al. [35] used the carbon nanotube core as a filler to prepare a composite. When the filler content was 1.94 wt.%, the thermal conductivity of the composite was 50 times that of the matrix. The above two fillers are metal and carbon fillers, respectively. Adding them to a composite can greatly improve the thermal conductivity of the composite but reduces the insulation performance. Therefore, for electrical equipment, like composite cross-arms, such fillers should be restricted, and the selection and modification of thermally conductive fillers should be studied in depth [36].

Currently, inorganic fillers have attracted attention owing to their excellent electrical insulation and their ability to improve other properties. They are mainly categorized into two: metal oxides and metal nitrides. The commonly used inorganic fillers are silicon nitride, boron nitride (BN), aluminum nitride (AlN), silicon oxide, and aluminum oxide (Al_2_O_3_). Among them, nitride fillers (such as silicon nitride, BN, and AlN) have excellent thermal and insulating properties, and they are preferred for thermally conductive insulating fillers [37,38,39].

Hong et al. [40] investigate the influence of relative composition and different sizes on the heat conduction path by designing a composite system of AlN and BN mixed fillers. When the relative composition of AlN and BN was 1:1 and the particle size was similar, the thermal conductivity was the largest because the interface thermal resistance and conductive network may be sensitive to the relative composition mode of AlN and BN. The influence of the relative size of the filler on the heat conduction path can be represented by a bimodal distribution curve. When the bimodal distribution is a continuous valley, the number of conductive networks increases and the contact area would be optimized, resulting in high thermal performance. Moradi et al. [41] found that the thermal conductivity of a composite increases with an increase in the particle size of BN when the volume fraction of BN is controlled. This shows the effect of particle size on the thermal conductivity. The difference in the thermal conductivity of composite materials due to different sizes of fillers is mainly attributed to the difference in the interface thermal resistance between the filler and the matrix. After certain treatments, the compatibility of the filler–matrix interface can be enhanced, improving the thermal conductivity of the composite to a certain extent [42].

**Figure 6:**
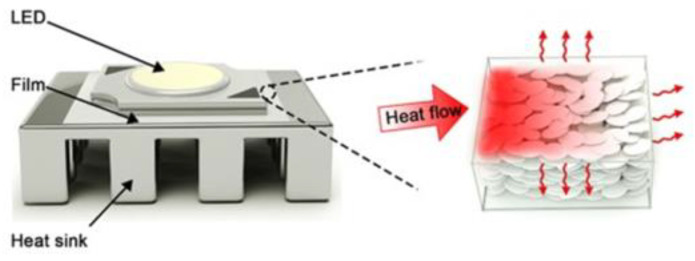
Schematic diagram of heat conduction in a BN film. Adapted with permission [44], Copyright 2021, Applied Nano Materials.

The shape of the filler is also one of the important factors affecting the thermal conductivity of a composite. The general 3D, 2D, and 1D fillers can form heat conduction pathways in different capabilities and methods. Sun et al. [43] compared the thermal conductivity of composites using hexagonal BN and hexagonal BN microspheres as fillers. With 40 wt.% hexagonal BN microspheres and hexagonal BN, the thermal conductivity of the composite material was 1.03 and 0.86 W·m^−1^·K^−1^, respectively. Recently, Wang et al. [44] obtained tangentially distributed BN nanosheets through self-assembly. The special structure has excellent insulation and thermal conductivity. When epoxy and BN films of the same specification were used as thermal interface materials, different heat dissipation effects were observed. The test device is shown in Figure 6. Finally, compared with commercial silicon wafers, the advantages of heat dissipation performance of BN films were analyzed through finite element simulation.

**Figure 7:**
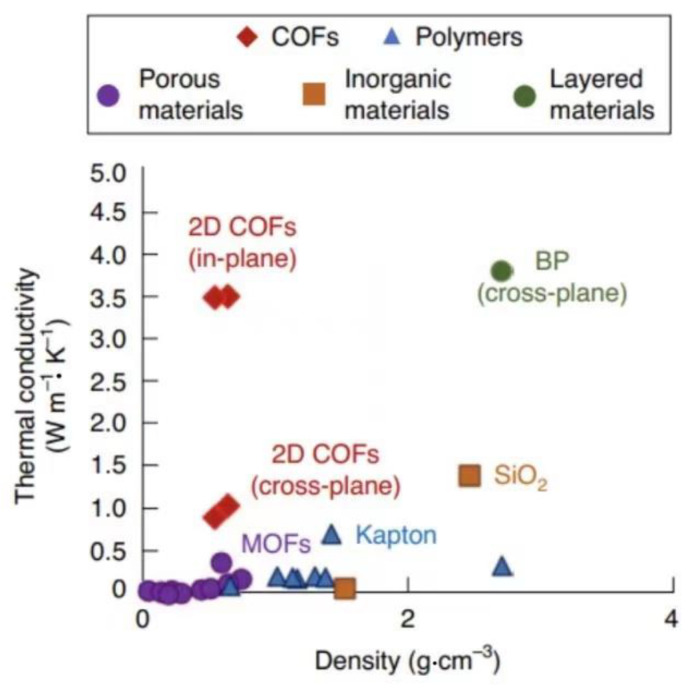
Relationship between the density and thermal conductivity of materials. Adapted with permission [45], Copyright 2021, Nature Materials.

Recently, 2D covalent organic frameworks (2D-COFs), which have the same properties, have been used in many fields. Evans et al. [45] synthesized 2D-COFs film by the template gel method. The thermal conductivity of the composite was as high as 1 W·m^−1^·K^−1^, and the dielectric constant was as low as 1.6. Compared with other organic or porous materials (Figure 7) 2D-COFs exhibit an extremely high thermal conductivity at the same density. In general, BN fillers have excellent thermal conductivity and insulation properties and can construct a heat conduction pathway inside composites after adjusting the microstructure. However, in practical applications, this type of filler is expensive and generally added only in a small amount, that is, it is added as an auxiliary filler together with other fillers to improve the thermal conductivity and insulation performance of composites.

**Figure 8:**
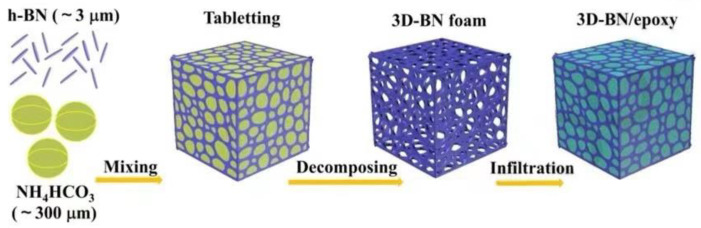
Schematic diagram of the formation process of 3D-BN/epoxy composite. Adapted with permission [50], Copyright 2020, Chemical Engineering Journal.

Considering the influence of the microstructure on the overall thermal conductivity of composites, a specially designed heat conduction path is constructed during the processing to reduce transmission resistance of phonons to obtain high thermal conductivity in a certain direction [46,47,48,49]. As shown in Figure 8, Xu et al. [50] constructed a 3D BN foam to achieve good thermal conductivity and insulation simultaneously. A self-supporting and pressure-enhanced 3D BN foam can be constructed by mixing BN powder and ammonium bicarbonate particles and then removing the ammonium bicarbonate by heat treatment. Then, the foam is immersed in a liquid epoxy mixture to obtain a 3D-BN/epoxy composite through a curing process. Chen et al. [51] mixed BN with a salt template at first to dissolve polyvinylidene fluoride (PVDF) as a binder in acetone to prevent the precipitation of BN (Figure 11). Then, they used acetone to evaporate and remove salt to form a BN network. Finally, they employed the vacuum-assisted impregnation method to infiltrate the epoxy resin into the stent to prepare the corresponding composites.

At present, the research on epoxy resin thermal conductive particle composites is mostly at the theoretical stage and has not been widely used in practical power engineering. The main reasons are that the poor dispersion of filler particles in the epoxy resin polymer matrix and the complex environment in actual power engineering (long-term low-temperature environment, high altitude low-voltage environment, magnetic field environment, salt spray environment, etc.) affect the long-term working stability of electrical equipment.

### 3.2. Filling Modification of Hollow Glass Microsphere

HGM is a closed-cell miniature sphere filled with CO_2_ gas. It has relatively good fluidity and dispersion in the matrix as it is small in size and its surface is smooth, which is essential for maintaining the uniform properties of composites [52]. HGM has many physical and chemical properties that other lightweight fillers cannot match since spheres have the smallest surface area among other shapes with the same volume. It is characterized by lightweight, high strength, stable performance, high melting point, high resistivity, and low thermal shrinkage coefficient, which enables HGMs to be widely used in composites. Matrices with HGM generally have good creep and heat resistance under certain processing technologies, which can also improve the mechanical properties of the composite without significantly increasing the weight, and simultaneously the dimensional stability and insulation of the composite are improved [21,31,53]. In the power field, HGM’s insulation and low thermal shrinkage coefficient can also be used to process and produce cable insulation material.

**Figure 9:**
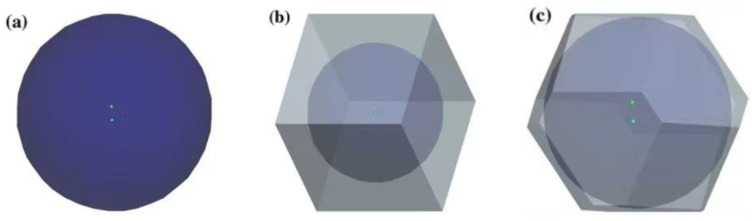
Corresponding HGM superimposed elements (**a**) Single; (**b**) Cubic; (**c**) Hexagonal HGM superimposed element. (**a**–**c**) Adapted with permission [54], Copyright 2018, MDPI.

Liu et al. [54] hypothesized that HGM in powder is mainly distributed in three configurations, namely, no-void, cubic dense, and hexagonal dense packing. A single HGM model is shown in Figure 9. It can be periodically repeated to form an entire composite. In the end, a 3D two-step layered calculation method is used to predict its effective thermal conductivity, and the results show that the hexagonal dense packing model can reflect the true distribution of the polymer better than the cubic dense packing and single-microsphere model.

**Figure 10:**
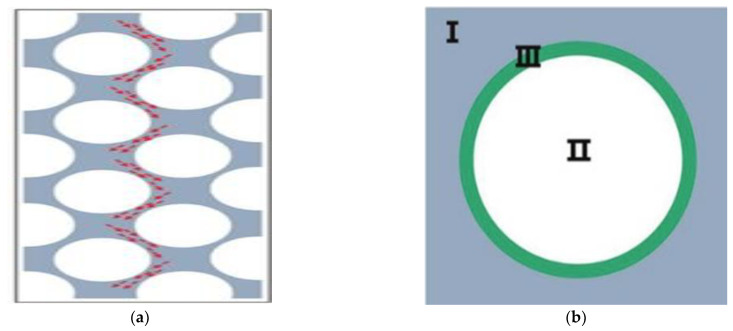
Microstructure between glass microspheres/matrix (**a**) Schematic of typical three-stage heat conduction; (**b**) Heat conduction of composite solids. (**a**,**b**) Adapted with permission [55], Copyright 2018, Advanced Materials.

Hu et al. [55] used a schematic diagram to show the plane between the glass microsphere/matrix (Figure 10a) and listed the heat transfer path between the composites (Figure 15). The contribution of the gas in HGM to the thermal conductivity is attributed to the collision of gas molecules in the void (stage II in Figure 10b) and between gas molecules and the solid wall of the interface (stage III). Heat transfer in solids (stage I) mainly depends on the molecular vibrations in the material. Heat transfer collisions at all stages can be described by dynamic theoretical models [56,57].

When there are some external factors, the original form of heat conduction will change. On the one hand, when the cavity of the glass microsphere is incomplete, the generated inorganic fragments can form additional continuous heat conduction paths to improve the thermal conductivity of the composite [58]. On the other hand, when the temperature is higher than the glass-transition temperature of the composite, a continuous network is obtained, which ultimately increases the heat transfer capacity [55,59].

**Figure 11:**
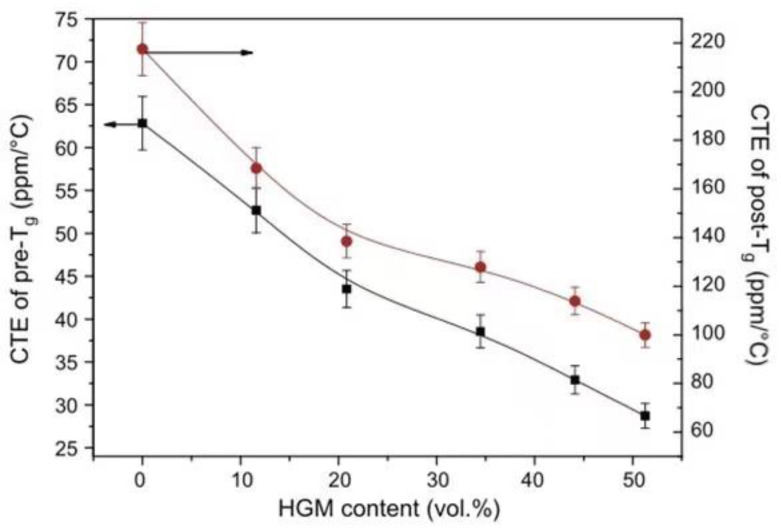
Thermal expansion coefficient of composites before and after the glass-transition temperature. Adapted with permission [62], Copyright 2008, Elsevier Ltd.

Considering thermal expansion, the significantly low CTE of glass microspheres and their interaction with epoxy resin are the main reasons for the decrease in CTE of the composite. The low CTE can enhance the thermal stability of the composite to a certain degree, and it is difficult to age under long-term light [60]. Pei et al. [61] established Table 1 based on experimental studies. The thermal expansion coefficient of a composite has a downward trend when the HGM ratio increases, and the performance of the composite is improved. Yung et al. [62] measured the CTE of a composite before and after the glass-transition temperature. As shown in Figure 11, the addition of HGM significantly changed the performance of the composite.

**Figure 12:**
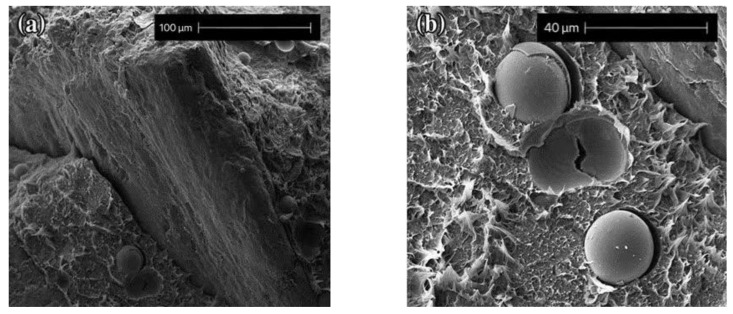
Interface diagram and partially enlarged figure of a composite without a coupling agent. (**a**) Mag = 460×; (**b**) Mag = 1415×. Adapted with permission [63], Copyright 2016, Springer.

**Figure 13:**
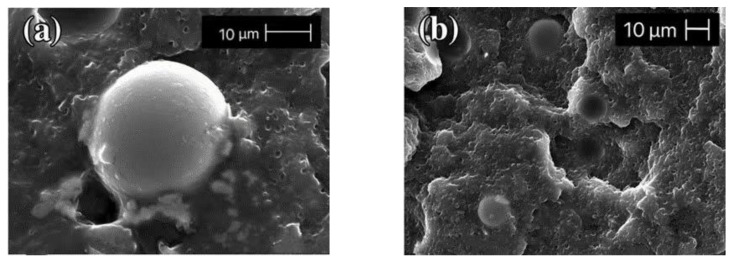
Interface diagram and partially enlarged figure of a composite with a coupling agent. (**a**) Mag = 1900×; (**b**) Mag = 1000×. Adapted with permission [63], Copyright 2016, Springer.

Tamara et al. [63] investigated HGM and Piasa fibers mixed with resin. Experiments showed that there is no great adhesion between HGM and the matrix in the absence of a coupling agent. This increases the thermal resistance of the filler/matrix interface. This weak interaction inevitably results in the poor electrical, mechanical, and thermal properties of the composite, and the heat transfer capability can be significantly enhanced by optimizing the filler–matrix interconnection. This is consistent with the experimental results of Wouterson et al. [64]. The microscopic morphology of the sample is shown in Figure 12 and Figure 13. It shows that it is necessary to modify the surface of HGM to improve the performance of the composite. Common surface modification methods include coupling agent modification, acid–base etching, plasma surface treatment, and elastomer-coating treatment [65].

The study on the thermal properties of epoxy resin-based syntactic foams was carried out from the perspective of different composites, and the related theory of glass bead filler was used to analyze the problems. The traditional research mode of simple blending was broken through, so that the study on thermal properties of epoxy composite foam could be solved from a new perspective. After modifying glass microspheres, the interface connection between HGM and the substrate can be changed from simple physical miscibility to a strong chemical bond. The microsphere and matrix are better connected [66,67,68], giving the composite unique physical and chemical properties. As in-depth research is conducted on microspheres, better epoxy-based composite foam insulation materials will be further explored.

### 3.3. Filling Modification of Negative CTE

Due to the porous structure, epoxy-based composite foams exhibit a higher CTE than most polymer materials. Close to room temperature, the linear expansion coefficient of epoxy-based foam is 40–80 ppm/K which is also much higher than that of common metallic and ceramic materials. Therefore, when such materials are used, there is a large CTE mismatch with other materials. Especially after many thermal cycles, the two materials generate greater stress at the contact surfaces, which can cause the instability of device performance [69].

NTE materials have the opposite characteristics of conventional “thermal expansion and cold contraction”; that is, materials with a negative average CTE within a certain temperature range. The NTE phenomenon can be divided into two types: phase transition and framework types. The phase transition type can be divided into atomic radius contraction and magnetic volume effect. In general, phase change materials have larger negative CTEs and narrower temperature ranges than frame-type materials [70,71]. When a composite material with a negative CTE filler is employed in composite cross-arms, the low thermal expansion would reduce the thermal mismatch stress and achieve high-precision control of thermal expansion, thereby controlling the generation and development of interface cracks. Although the NTE phenomenon has been observed for a long time, the negative expansion phenomena of most materials, such as ice, quartz, Si, and zeolite, are restricted to a very narrow temperature range [72,73,74,75,76]. In addition, NTE materials have anisotropic thermal shrinkage and cold expansion when the temperature is too high or too low, which limits their applications.

**Figure 14:**
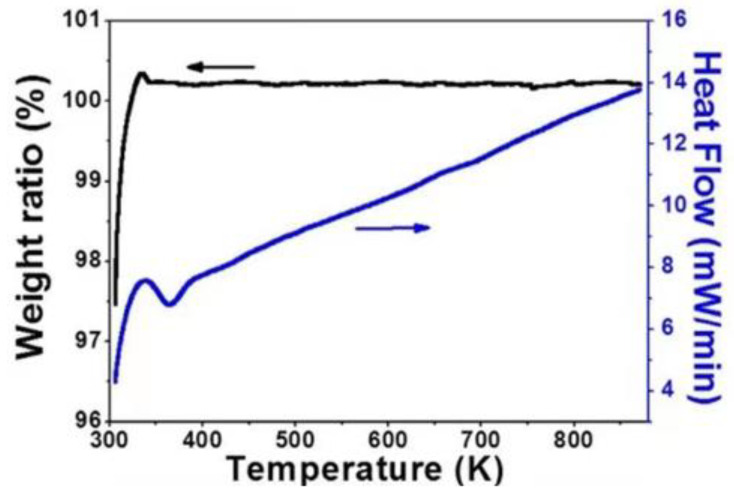
Thermogravimetric differential scanning calorimetry curve. Adapted with permission [78], Copyright 2015, Scientific Reports.

Since the discovery of ZrW_2_O_8_ in frame structure materials, NTE materials have attracted widespread attention and developed rapidly [77,78]. Since then, much more NTE materials have been discovered. Sleight found materials with strong NTE effects, a wide temperature range, and isotropic properties in tungsten oxides [79], making the material attract global attention. The CTE of the composite can be significantly reduced when a small amount of NTE material is added to the composite. Ge et al. [80] reported a new material with a molecular formula of ZrScMo_2_VO_12_. This material has overcome the temperature limitation of general NTE materials and could have excellent continuous NTE characteristics in a wide temperature range. The moisture absorption rate within the studied temperature range is close to zero. The thermogravimetric/differential scanning calorimetry curve from room temperature to 873 K is shown in Figure 14 (hygroscopicity causes abnormal changes in the linear thermal expansion curve) [81]. Zhang et al. [82] found that when NTE material is used as a thermal expansion inhibitor to compound with epoxy resin, only 22.5 vol.% of Mn_0.983_CoGe is required to completely suppress the positive thermal expansion of epoxy resin. Notably, the thermal conductivity of composites has also increased significantly. For example, the thermal conductivity of Mn_0.983_CoGe/epoxy resin composite with a filling amount of 30 vol.% is almost twice that of pure epoxy resin at room temperature, and it is increased three times to about 150 K.

Compared with other fillers, NTE fillers are very scarce, and common chemical properties, such as NTE coefficient, thermal expansion anisotropy, and mechanical or electrical insulation performance, are within a limited range. Therefore, only very limited NTE materials are used as thermal expansion compensators in practical applications [80,83]. When using small-sized fillers, attention should be paid to the unique chemical properties of NTE materials to avoid chemical deterioration of the matrix and filler during the formation of the composite material, thus causing the filler to lose its NTE properties [84]. In addition, research on NTE materials not only focuses on developing new NTE materials but also adjusting their CTE to meet the complex engineering needs of composite cross-arms. To study the essential mechanism of isotropic NTE materials, there is a need to develop more NTE fillers with a wide temperature range and stable performance.

### 3.4. Fiber Filling Modification

Compared with particulate fillers, the strength and elastic modulus of fiber materials have great advantages. First, the fiber fillers with high thermal conductivity can not only increase the thermal conductivity of the composite but also serve as the main carrier in the composite. When the composite is subjected to an external force, the force is distributed to the internal fibers so that the load is evenly distributed, and its mechanical strength and modulus will greatly exceed those of conventional bulk polymers [85]. In addition, with an increase in fiber content, it is easy to form a larger heat conduction network in the matrix so that the heat flow circulates along the radial direction of the fiber, and this improves the heat conduction efficiency of the material along the radial direction of the fiber. Therefore, a combination of fiber and matrix results in a composite with high thermal conductivity and low weight [86]. In terms of thermal expansion, the generation of thermal stress may cause the bending of fibers, resulting in fiber debonding and interfacial crack propagation (even when the fiber has been modified), which greatly affects the thermal, electrical, and mechanical properties of the composite. Factors affecting the thermal conduction and thermal expansion of composites have been extensively studied, including fiber volume fraction, orientation arrangement, cross-sectional area, microstructure, and modification effects [87].

In the first section, we mentioned the overall thermal conductivity of composites can be improved by the ultra-high thermal conductivity of BNs, such as building a 3D network [88,89,90,91,92,93]. Hou et al. [94] employed this principle to make BNs fibers. BNs and carbon fibers were dispersed in a solvent, and after a series of high-temperature reactions, aluminum borate/BNs fibers were obtained. The test results show that the treated BNs significantly enhanced the thermal conductivity of the composite. Guoliang et al. [95] obtained the axial and radial thermal conductivity of a single fiber through indirect measurement of composite materials and establishing models and theoretical calculations because the measurement of the thermal conductivity of a single fiber is difficult. They expressed the prospect of heat conduction in different arrangements of the fibers. On the one hand, the thermal conductivity of the composite increased as the fiber volume fraction increased. On the other hand, the fiber fineness greatly affects the thermal conductivity. Ronca et al. [96] obtained similar results after experimental analysis of fiber samples.

**Figure 15:**
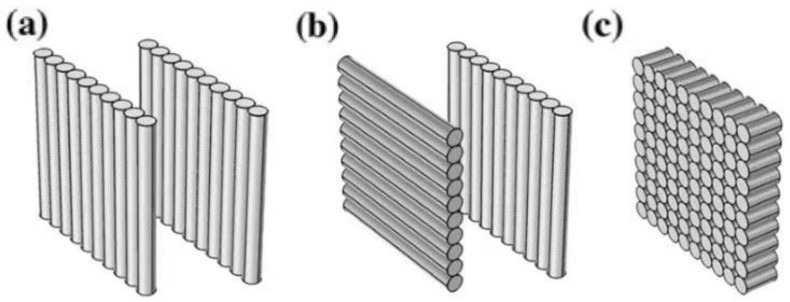
Different arrangement directions of fibers. (**a**) pattern 1 with films overlapped along the same direction; (**b**) pattern 2 with films overlapped and rotated by 90°; (**c**) pattern 3 with fiber oriented to the out-panel direction. (**a**–**c**) Adapted with permission [95], Copyright 2019, Applied Thermal Engineering.

Tan et al. [97] studied the effect of different fiber orientations on thermal conductivity. They divided the fiber arrangement direction into three categories, as shown in Figure 15. Figure 15a is overlapped and has the same fiber arrangement direction. In Figure 15b, the fibers are rotated by 90°. In Figure 15c, all the fibers are arranged toward the outer plate. Comparing the final verification with the volume fraction of the fiber, the orientation of the fiber is a very important factor that affects temperature distribution [98,99].

Recently, the wide application of fibers in composite cross-arm has promoted the development of manufacturing processes for composite cross-arms. When the composite cross-arm is used in a low-temperature or low-temperature alternating environment, the fiber composite will be damaged, decreasing the safety of the composite. The thermal expansion makes the boundary between the matrix and the fiber prone to damage. Fibers would be better used in electrical equipment after being researched and explored further in future studies as a reinforcing phase of composites.

## 4. Conclusions and Prospect

This paper presents the latest developments in thermally conductive particles, HGM, negative CTE-material, and fiber filling modifications considering thermal conductivity and expansion performance to determine a suitable modification method for preparing the inner filling material for composite insulation cross-arms. Recently, with more in-depth research, novel types of composites for preparing composite insulation cross-arms exhibit several high-quality properties under different filler modifications. The advantages are obvious, especially in terms of being lightweight, high strength, having good insulation performance, high thermal conductivity, and low thermal expansion. However, there are still many challenges in the compounding process. Such challenges are not limited to the types of substrates and fillers but also the complicated structural connections, chemical reactions, heat conduction, and interfacial problems. These have hindered the realization of the envisioned production mechanism. In this regard, we propose the following prospects:

Under high loads, the load on epoxy resin-based composite foam insulation materials modified by fiber filling is distributed along the fiber, and the load distribution is more uniform. Further research on fiber/matrix interfaces is required because the actual application environment of the composite insulation cross-arms is complicated, and the interface-coupling strength between the fiber and the matrix still needs to be improved.

The mechanical properties of epoxy resin-based composite foam insulation materials with high filling decreases with an increase in the filler content, which cannot guarantee the quality of actual engineering applications. Therefore, there is a need to investigate novel low-filling-content and high-modification-efficiency fillers by exploring theoretical models of thermal characteristics to ensure the overall quality of composite insulation cross-arms.

High-efficiency transmission of curing reaction heat is important for modifying the thermal properties of epoxy resin-based composite foam insulation materials. Therefore, in addition to enhancing the thermal characteristics of the inner filling material of composite insulating cross-arms, the difficulty in modifying the thermal characteristics can be fundamentally reduced by controlling the curing reaction heat, which has great significance to the engineering production of composite insulating cross-arms.

## Figures and Tables

**Table 1:** Thermal expansion coefficient of HGM/epoxy resin composite. Adapted with permission [61], Copyright 2020, Chinese Journal of Colloid & Polymer.

HGM	0%	2%	4%	6%	8%
Thermal expansion coefficient	4.39 × 10^−5^	2.79 × 10^−5^	2.75 × 10^−5^	2.79 × 10^−5^	2.39 × 10^−5^

## Data Availability

Not applicable.
